# Structure and Properties of Cast Ti-Al-Si Alloys

**DOI:** 10.3390/ma14040813

**Published:** 2021-02-08

**Authors:** Anna Knaislová, Pavel Novák, Jiří Linhart, Ivo Szurman, Kateřina Skotnicová, Jan Juřica, Tomáš Čegan

**Affiliations:** 1 Department of Metals and Corrosion Engineering, University of Chemistry and Technology, Prague, Technická 5, 166 28 Prague 6, Czech Republic; knaisloa@vscht.cz (A.K.); linhartj@vscht.cz (J.L.); 2 Department of Non-Ferrous Metals, Refining and Recycling, Faculty of Materials Science and Technology, VSB—Technical University of Ostrava, 17. listopadu 15, 708 33 Ostrava-Poruba, Czech Republic; ivo.szurman@vsb.cz (I.S.); katerina.skotnicova@vsb.cz (K.S.); jan.jurica@vsb.cz (J.J.); tomas.cegan@vsb.cz (T.Č.)

**Keywords:** intermetallics, casting, Ti-Al based alloys

## Abstract

Intermetallic compounds based on Ti-Al- (Si) are attractive materials with good thermal stability and low density. However, the production of these materials is quite complicated. Partially modified conventional methods of melting metallurgy are most often used due to availability, possible high productivity, and relatively low production costs. Therefore, some technologies for the production of intermetallics based on Ti-Al are currently available, but with certain disadvantages, which are caused by poor casting properties or extreme reactivity of the melt with crucibles. Some shortcomings can be eliminated by modifying the melting technology, which contributes to increasing the cost of the process. The work deals with the preparation of Ti-Al-Si intermetallic compounds with different contents of aluminum and silicon, which were produced by centrifugal casting in an induction vacuum furnace Linn Supercast-Titan. This process could contribute to the commercial use of these alloys in the future. For this research, the TiAl15Si15(in wt.%) alloy was selected, which represents a balanced ratio of aluminides and silicides in its structure, and the TiAl35Si5 alloy, which due to the lower silicon content allows better melting conditions, especially with regard to the melting temperature. This alloy was also investigated after HIP (“Hot Isostatic Pressing”) treatment.

## 1. Introduction

Extensive testing of alloys based on intermetallic compounds of the Ti-Al system, which has been carried out since the 1980s, has enabled their commercial application in the aerospace and automotive industries. TiAl alloys are currently used commercially, mainly for the production of turbochargers and aircraft engine blades [1,2,3].

Four classes of alloys based on intermetallic compounds of the Ti-Al system are presented in [4]. The first generation is based mainly binary alloys with different aluminum contents in the range of 42–48 at.%. These alloys show very low ductility and reduced resistance to creep and oxidation at high temperatures [1,4]. In order to improve the properties, second generation TiAl alloys have been developed. Representatives of this class include TiAl48Mn2Nb2 and TiAl48Cr2Nb2 alloys, which are still commercially applied today to the blades of low-pressure parts of a combustion turbine. Additionallyincluded ones are TiAl47W2Si0.5 alloy and modified TiAl47W2Si0.5B0.5 alloy. During the development of second-generation TiAl alloys, the positive effect of boron on the alloy structure was demonstrated. Boron alloying has proven to be an effective method for preventing the uncontrollable growth of individual alloy grains during heat treatment and thermal exposure during service [1,4]. Due to the persisting limitation in terms of ductility, a third generation of TiAl alloys has been developed. This generation of alloys has been developed primarily for hot forming technology. These TiAl-based alloys are highly alloyed mainly with niobium and small amounts of carbon and boron. Due to their excellent mechanical properties and resistance to high temperature oxidation and creep up to 800 °C, they become suitable materials for various components in gas turbines, jet and automobile engines [3,4]. Due to the growing demands on these alloys with regard to aerospace components, which should be able to withstand ever-increasing temperatures, the latest fourth generation of TiAl-based alloys is being developed, represented by Ti46Al8Ta (at.%) alloy. This compound is alloyed with tantalum, which significantly reduces the diffusion in the material for its strengthening, even at low cooling rates [4].

More than ten years ago, TiAl48Nb2Cr2 alloy was first commercially applied to the GEnx engine’s low-pressure turbine blades. The lower centrifugal load of the lighter TiAl blades reduces the total weight of the disc, which contributes to a significant part of the saving of the total weight of the motor, up to 100 kg. Obtained knowledge from the operation show that the GEnx engine delivers a 20% reduction in fuel consumption, a 50% reduction in noise and an 80% reduction in NO_X_ emissions compared to previous engines in the same series, which use conventional nickel alloy blades. Today, approximately 190 000 TiAl blades are applied to the low-pressure parts of combustion turbines fitted to Boeing 787-s and 747-8s aircraft engines. Furthermore, this application of TiAl alloy is also planned for new LEAP engines [1,2,3]. In addition to the GEnx engines already mentioned, the application of stabilized β-TiAl alloy for the production of LPT blades for PW1100 G engines has now expanded. Rolls-Royce has also announced the application of TiAl blades for low pressure combustion turbine parts for its future engines. Furthermore, the application of TiAl alloys is considered for covers and blade holders and turbine dampers. To date, however, the TiAl blades of the low-pressure parts of the combustion turbine of the GEnx engine are the only large-scale commercial application in the aerospace industry [1,2].

One of the most successful applications of TiAl alloys from the point of view of the automotive industry is the production of turbochargers for the Japanese automotive company Mitsubishi Motors Inc. These turbochargers are successfully used for Mitsubishi Lancer production cars. In 2002, forged exhaust valves for internal combustion engines made of γ-TiAl alloy were applied to Formula 1 cars, which today do not meet the required standards and therefore had to be withdrawn. Due to the favorable properties of alloys, such as low density, high strength and fatigue resistance, there is an effort to modify the production technology and re-application of exhaust valves for the automotive industry [5,6].

The above commercial and future applications show a strong interest in the further development and subsequent application of these alloys in the aerospace and automotive industries. Extensive research into TiAl-based alloys aims to reduce engine weight and improve engine performance. The current state of production technology of TiAl intermetallics is the result of several decades of significant research and development in academia and industry around the world. Today, research is mainly concerned with improving the alloy itself and production technologies to achieve the required properties adapted to specific applications, and TiAl-based alloy components will be more competitive in the future and their potential can be fully exploited [2,7].

The processing technologies of intermetallics, which are considered or already applied in industrial praxis, include most commonly the methods of melting metallurgy. For the processing of intermetallics, the ExoMelt process had been developed [8], tested for Fe-Al intermetallics and could be applicable also for the processing of Ti-Al based alloys. This process comprises a method of charging initial components in the furnace in order to utilize the heat generated by the reactions between the aluminum and transition metals, which form the intermetallics. Otherwise, vacuum induction melting has become a standard in processing of Ti-Al based intermetallics [9,10,11,12,13,14]. The material solution of the melting crucible is also a problem in the case of Ti-based intermetallics due to extreme reactivity of the melts [15]. The ceramics based on yttria (yttrium oxide) is widely applied [16], but even in such a case the melt could be contaminated by yttrium. Calcium zirconate crucibles could be a solution [17]. However, several teams succeeded with using graphite as the crucible material for Ti-based intermetallics [18,19]. Due to a poor castability of these materials, centrifugal casting is often applied [20,21,22]. In order to heal the internal porosity of the castings, hot isostatic pressing (HIP) is required in some cases [23,24].

This work deals with the addition of silicon into the TiAl alloys. Silicon is a suitable alloying element that improves the resistance of intermetallics TiAl to oxidation and creep at high temperatures. It has a very low solubility in the given intermetallic and therefore forms a stable silicide Ti_5_Si_3_ with titanium. It is an intermetallic phase with a high melting point (2130 °C) and a low specific gravity. Furthermore, silicon has a positive effect on the formation of an adhesive and compact oxide layer, which in the case of intermetallics based on Ti-Al-Si consists of a mixture of TiO_2_ and Al_2_O_3_ and SiO_2_, which heals unwanted pores. For these reasons, intermetallics are resistant even at higher temperatures [25,26,27,28]. Recently developed Ti-Al-Si alloys have been prepared by powder metallurgy processes, especially reactive sintering [26,29] and the technology consisting of mechanical alloying and spark plasma sintering [30]. However, these methods, even though they enable rapid preparation and almost free choice of the chemical composition of the product, are not so suitable for mass industrial production. This paper aims to test the vacuum induction melting with centrifugal casting as the processing route of these materials and describes the properties of the product.

## 2. Materials and Methods

Experimental alloys TiAl15Si15 and TiAl35Si5 (wt.%) were prepared by vacuum induction melting with centrifugal casting (CC). For the preparation, the Linn Supercast-Titan device was used. The melting operations were performed at VSB-Technical university of Ostrava, department of Non-ferrous metals, refining and recycling. As raw/input materials, Ti (grade 2, round bar, diameter 10 mm, height up to 60 mm), Al (99.99 wt.%, pieces up to 20 mm × 20 mm × 20 mm) and Si (99.99 wt.%, pieces up to 10 mm × 10 mm × 10 mm) were used. The total volume of the melt was 100 cm^3^. Used graphite crucible was made of isostatic pressed graphite—SGV5-G B 527 XN. For each alloy, a separate crucible was used. Graphite casting form (inner diameter 20 mm, height 225 mm) with no-preheating was used. Prior to melting, the device was evacuated several times and filled with Ar (99.9999%). Melting was realized under low pressure of protective gas. Rotating speed during casting was 400 rpm. By this way, cylindrical samples were obtained. The selected TiAl35Si5 alloy was also subjected to hot isostatic pressing (HIP), which took place in a furnace at a temperature of 1260 °C and a pressure of 190 MPa for 4 h with heating at 10 °C/min. This technology was chosen for minimizing the internal porosity of the casting.

Cast alloys TiAl15Si15 and TiAl35Si5 were subjected to X-ray diffraction analysis using a diffractometer PANalyticalX’Pert Pro ((PANalytical, Almelo, Netherlands) followed by evaluation in X’PertHighScore 3.0 software package (PANalytical, Almelo, Netherlands) using PDF-2 2018 database to identify the phase composition. Metallographic cuts were prepared to study the microstructure of experimental alloys. Samples were etched by Kroll’s agent (5 mL HNO_3_, 10 mL HF, 85 mL H_2_O) due to the observing of the microstructure with a Nikon Eclipse MA200 light microscope (Nikon, Tokyo, Japan) using the NIS Elements (Laboratory Imaging, Prague, Czech Republic) program. The individual phases and chemical composition were examined using a TESCAN VEGA 3LMU scanning electron microscope in backscattered electrons regime (TESCAN, Brno, Czech Republic) with an Oxford Instruments X-max 20 mm^2^ EDS analyzer (Oxford Instruments, High Wycombe, UK). The porosity of the alloys was determined by the image analysis of the optical micrographs by ImageJ 1.53 software.

From the mechanical tests, Vickers hardness with a load of 5 kg (HV 5) was measured from 10 indention in each sample. Tests of compressive strength were conducted by the means of the universal testing device LabTest 5.250SP1-VM (produced by LaborTech, Opava, Czech Republic). Values of ultimate tensile strength in compression were determined from the measured loading curves. Tribological properties was determined by the TRIBOtester ball-on-disc tribometer (Tribotechnic, Clichy, France) with subsequent evaluation in the TRIBOtechnic program. The wear tests took place at a load of 5.0 N, with a total path of 20,000 mm and a displacement speed of 15 mm/s, which was performed by a spherical body of Al_2_O_3_ with a diameter of 6 mm as the static partner. The eccentricity of the device was set to 5 mm and for this reason the abrasion area on the sample reached a length of 10 mm. The whole measurement took place at room temperature and the result was the measured wear rate of the sample and the average coefficient of friction. The resulting wear tracks were subsequently examined using the scanning electron microscope with the EDS analyzer described abovein order to approach the wear mechanism.

High-temperature properties of alloys were investigated by cyclic oxidation tests at 800 °C and 1000 °C for a total length of 400 h. The length of one oxidation cycle was 50 h (8 cycles per 50h). At the end of each cycle, the samples were removed from the furnace. After spontaneous cooling in air, they were weighed by an analytical scale Pioneer Plus (Ohaus, Parsippany, NJ, USA) with an accuracy of 0.0001 g and placed back in the furnace at the set test temperature. The oxidation rate was determined as the weight gain that resulted from the formation of an oxide layer on the surface of the individual experimental alloys. The phase composition of the formed oxide layers was determined by X-ray diffraction analysis using a PANalyticalX´Pert Pro diffractometer and the microstructure of the oxide layers was examined using SEM-EDS.

## 3. Results

### 3.1. Microstructure and Phase Composition

**Figure 1:**
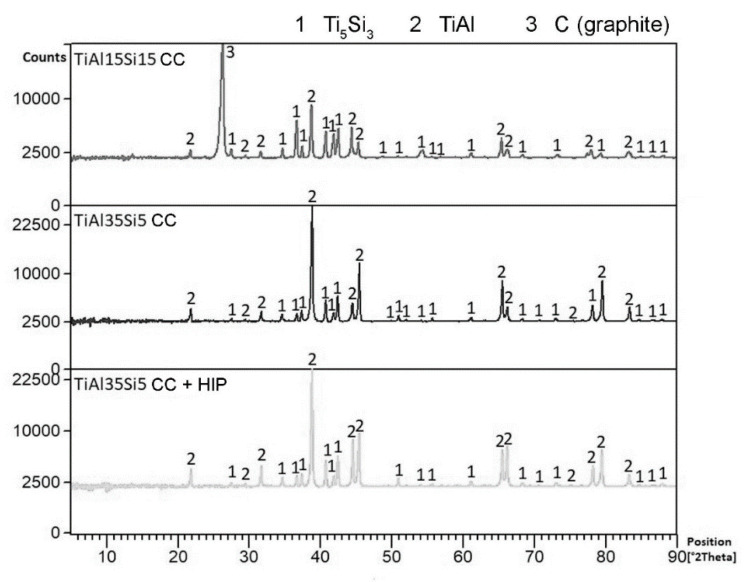
XRD patterns of the tested alloys.

**Figure 2:**
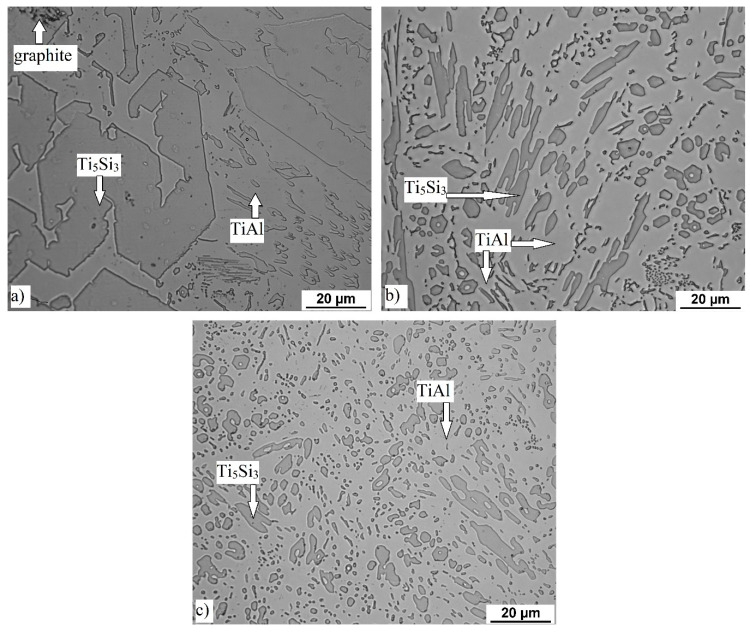
Optical micrographs of the tested alloys: (**a**) TiAl15Si15 in as-cast state, (**b**) TiAl35Si5in as-cast state, (**c**) TiAl35Si5 after hot isostatic pressing (HIP).

Microstructure of both tested alloys consists of titanium silicide (Ti_5_Si_3_) particles in titanium aluminide (TiAl) matrix, see Figure 1 and Figure 2. In addition to these phases, graphite was also detected in TiAl15Si15 alloy (Figure 1) as a result of required overheating of this high-silicon alloy and corresponding interaction of graphite with the melt. The size (equivalent diameter) of silicide particles reaches approximately 50 and 10 in cast TiAl35Si5 and TiAl15Si15 alloys, respectively. The silicide particles exhibit mostly sharp-edged morphology in both alloys in as-cast state. Hot isostatic pressing of the TiAl35Si5 casting led to partial spheroidization of the Ti_5_Si_3_ particles, leading to round shape of the silicide. The porosity of all tested alloys determined by image analysis is very low. As-cast alloys reach the porosity of about 1 vol. %, after HIP the porosity decreased to 0.7 vol. %.

### 3.2. Mechanical and Tribological Properties

The hardness of the Ti-Al-Si alloys increases with the amount of silicon (Table 1). TiAl15Si15 alloy reaches a higher hardness than TiAl35Si5 alloy. Hot isostatic pressing has not had an effect on the hardness of the material, TiAl35Si5 alloy has the same hardness before HIP and after HIP. Values of ultimate compressive strength increase with the amount of aluminum and HIP has not an effect on these values. TiAl35Si5 alloys have the highest values due to its structure, which is largely formed by relatively tougher aluminides TiAl.

**Figure 3:**
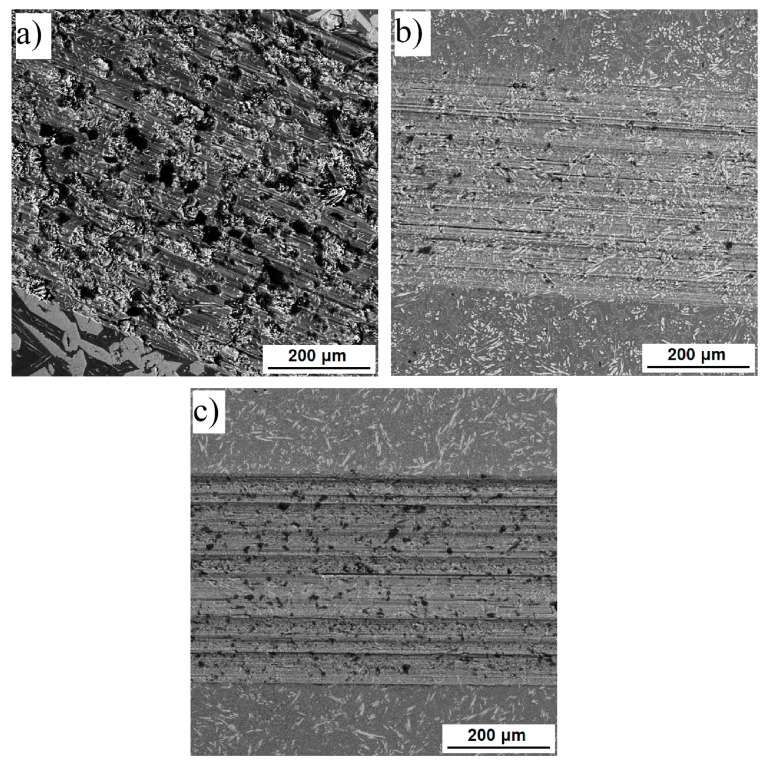
Morphology of the wear tracks (SEM-BSE) on: (**a**) TiAl15Si15 in as-cast state, (**b**) TiAl35Si5 in as-cast state, (**c**) TiAl35Si5 after HIP.

Tribological properties were tested under the conditions of a dry sliding wear against alumina as the static friction partner. The results indicate that the friction coefficient, as well as the wear rate, are influenced by the amounts of silicon and aluminum in the alloy. The high-silicon material (TiAl15Si15) exhibits the highest friction coefficient (Table 2) and wear rate. Observation of the wear track by scanning electron microscope in backscattered electrons regime revealed that this alloy tends to a strong removal of the silicide particles from the surface due to their brittle nature (Figure 3a). This phenomenon can be seen also in the case of the low-silicon (FeAl35Si5) material, but in much lower extent (Figure 3b,c). The wear at all of the tested materials is mostly abrasive, where the extracted hard silicide particles act as abrasive. There are no visible signs of oxidation of the material in the wear track. The influence of HIP processing on the friction coefficient, wear rate, and mechanism of the wear damage is almost negligible.

### 3.3. High-Temperatute Properties

**Figure 4:**
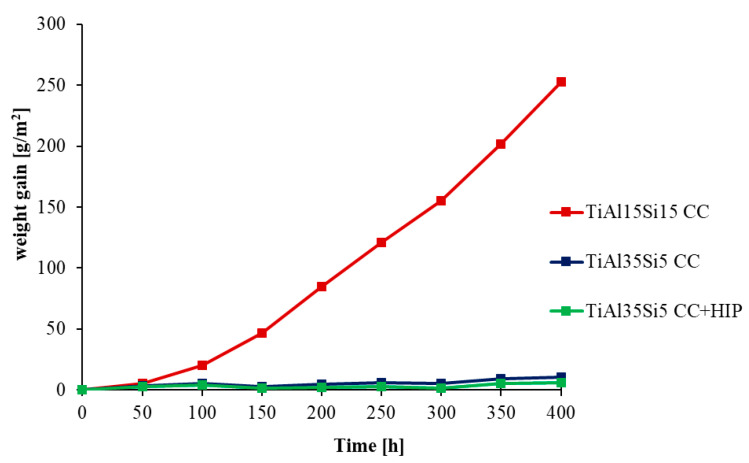
Dependence of total weight gain of Ti-Al-Si alloys (including delaminated oxides) on cyclic oxidation duration (800 °C; 400 h).

**Figure 5:**
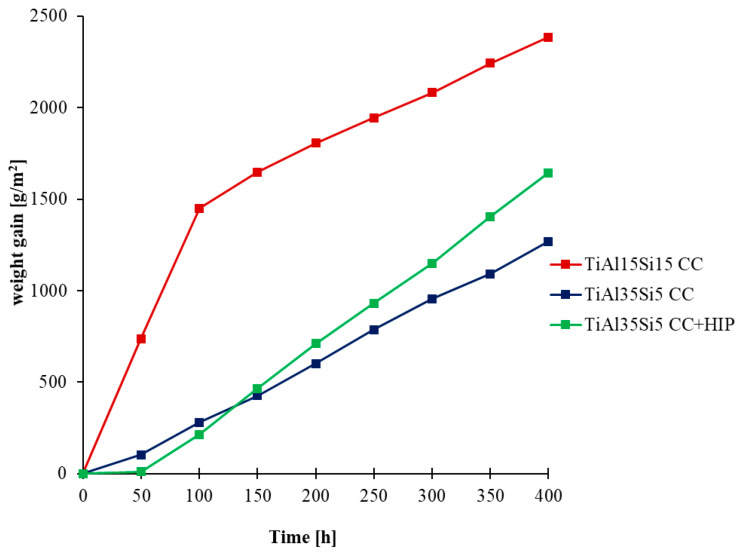
Dependence of total weight gain of Ti-Al-Si alloys (including delaminated oxides) on cyclic oxidation duration (1000 °C; 400 h).

**Figure 6:**
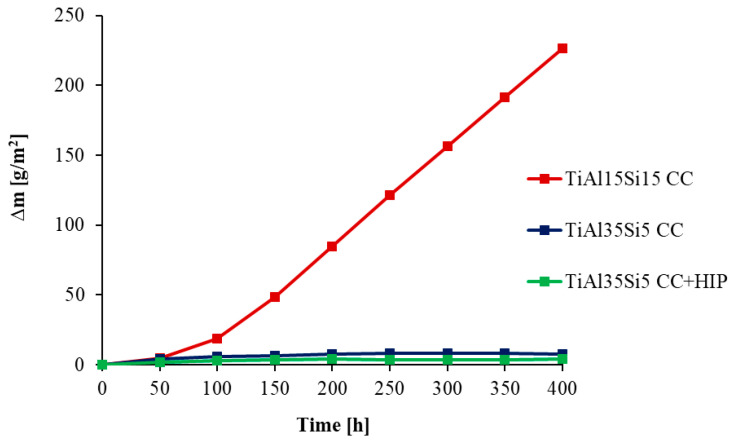
Dependence of the weight of delaminated oxides of Ti-Al-Si alloys on the time of cyclic oxidations (800 °C; 400 h).

**Figure 7:**
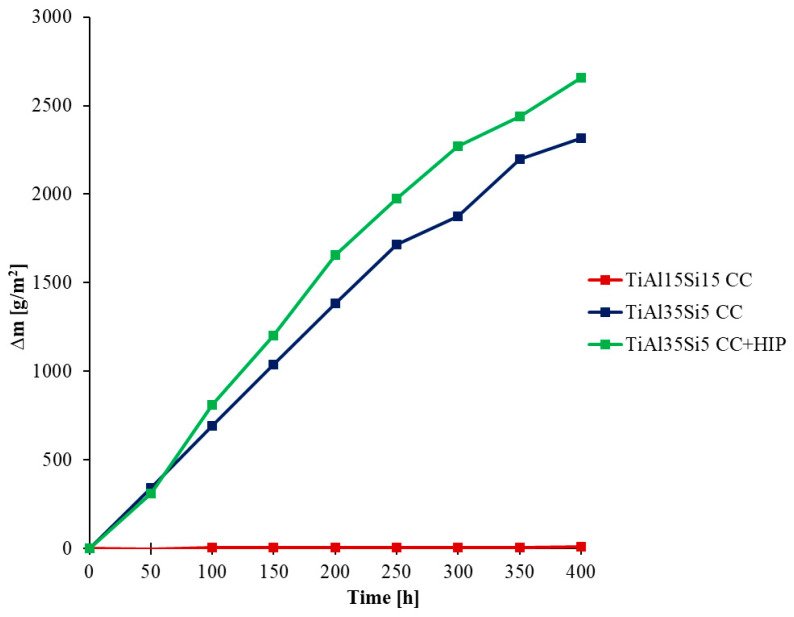
Dependence of the weight of delaminated oxides of Ti-Al-Si alloys on the time of cyclic oxidations (1000 °C; 400 h).

The oxidation rate was determined from the weight gain obtained due to the formation of oxides on the surface of the base material during thermal exposure. The weight gain can be observed on the kinetic curves in Figure 4 for 800 °C and in Figure 5 for 1000 °C, when the studied alloys were weighted, including scaled-off (delaminated) oxides. For a more detailed view of the delamination of oxide layers, kinetic curves were generated only for delaminated oxides, in Figure 6 for 800 °C, and in Figure 7 for 1000 °C.

Cyclic oxidation tests at 800 °C showed good oxide layer adhesion for all alloys. The highest increase in oxidation rate was shown by the TiAl15Si15 alloy at both process temperatures. This alloy contained large sharp-edged silicide particles in its structure and in particular it was contaminated by carbon. After cyclic oxidation tests of this alloy, there was most likely a better oxygen permeability to the base material during exposure due to the diffusion of carbon from the structure at a higher temperature. For this reason, there was a constant growth of the oxide layer and continuous oxidation of the whole material, which was demonstrated for this alloy after cyclic oxidation tests at a temperature of 1000 °C, when the whole base material was completely oxidized. The resulting oxide layer is porous, but even at a temperature of 1000 °C it did not undergo massive delamination (Figure 7). In the case of cast alloys TiAl35Si5 and TiAl35Si5 after HIP, the results of cyclic oxidation tests at 800 °C showed the formation of a thin and well-adhering oxide layer, which protected the base material from subsequent oxidation. The weight gain was practically zero throughout the oxidation tests (see Figure 4). The formation of a stable oxide layer with protective effects results in a decrease in the oxidation rate due to the slowing down of the diffusion of oxygen through the oxide layer. In the cyclic oxidation tests, which took place at a temperature of 1000 °C, there was already an increase in the oxide layer with insufficient protective effect, and therefore a massive delamination of this layer followed in both TiAl35Si5 alloys, which allowed further oxidation of the base material. Poor adhesion of the protective layer can be detected in Figure 5. Alloys show repetitive parabolic growth of the oxide layer. Due to the insufficient protection of the base material by the respective surface oxides, the oxidation rate is controlled by the chemical reaction of oxygen with the surface of the material. The non-compactness of the layer during this exposure is partly due to the insufficient amount of silicon in the alloys.

**Figure 8:**
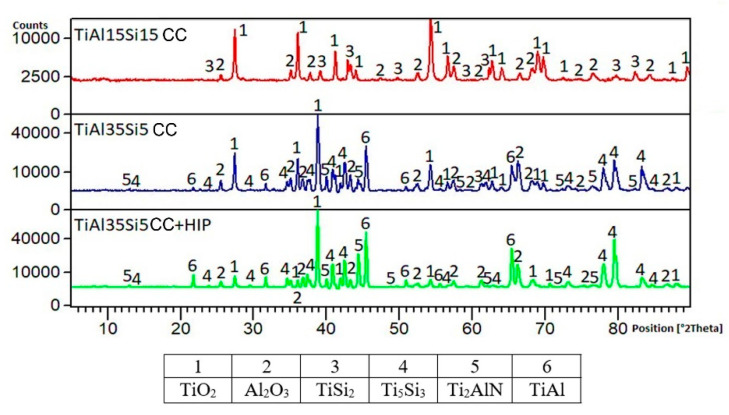
Phase composition of the surface oxide layer of Ti-Al-Si alloys after cyclic oxidation at 800 °C.

**Figure 9:**
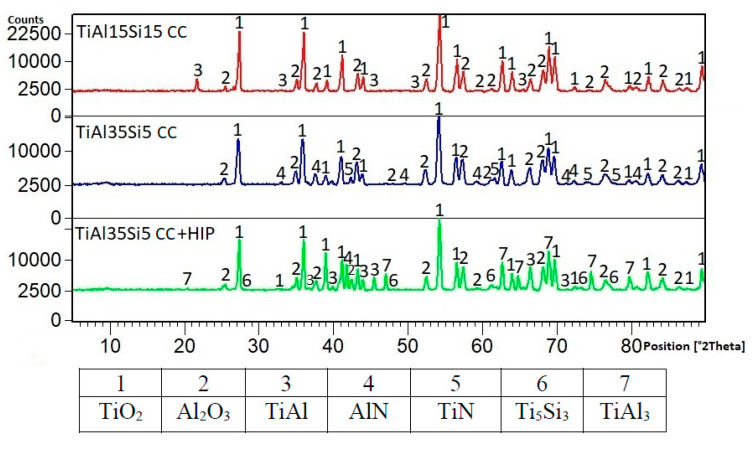
Phase composition of the surface oxide layer of Ti-Al-Si alloys after cyclic oxidation at 1000 °C.

The phase composition of the formed oxide layers of the studied Ti-Al-Si alloys was determined after cyclic oxidation tests by X-ray diffraction analysis. The presence of TiO2 and Al2O3 oxides, which formed the major components of the surface oxide layer, was proved in all alloys. After thermal exposure at 800 °C (Figure 8), the presence of titanium silicide was further presented in Ti-Al-Si alloys produced by centrifugal casting. In the case of TiAl15Si15 (CC) alloy, a higher TiSi_2_ silicide was detected on the surface, in contrast to TiAl35Si5 (CC) alloys, where the brittle phase of Ti_5_Si_3_ silicide was present. The surface layer of TiAl35Si5 (CC) and TiAl35Si5 (CC+HIP) alloys was further supplemented with a Ti_2_AlN phase, which forms an intermediate layer between the base material and the resulting oxide layer and further increases its adhesion. After thermal exposure at 1000 °C (Figure 9), there was no significant change in the phase composition of the cast Ti-Al-Si alloys compared to the previous results. In the case of the TiAl35Si5 (CC) alloy, the AlN and TiN phases were further detected, in which the same effect can be expected as in the mentioned Ti_2_AlN phase. In the cast HIP-treated TiAl35Si5 alloy, the presence of a higher titanium aluminide TiAl_3_ was detected, which was probably due to the increased concentration of aluminum in the surface layer of the alloy during thermal exposure.

**Figure 10:**
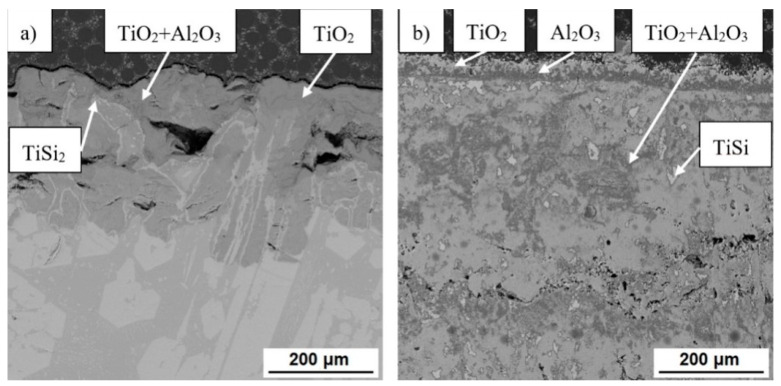
Microstructure (SEM-BSE) of oxide layers of TiAl15Si15 alloy, prepared by centrifugal casting, after cyclic oxidation at (**a**) 800 °C and (**b**) 1000 °C for 400 h.

**Figure 11:**
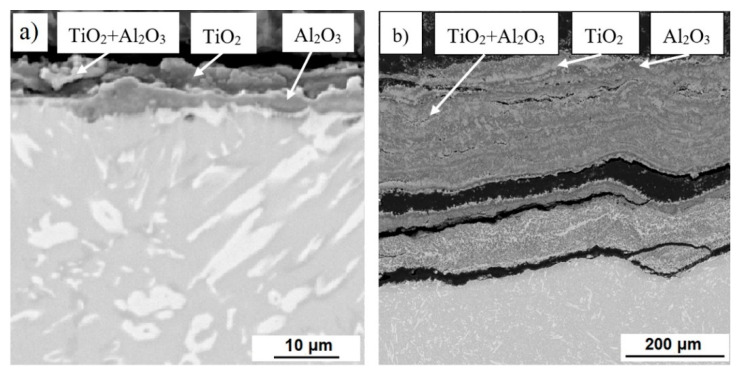
Microstructure (SEM-BSE) of oxide layers of TiAl35Si5 alloy, prepared by centrifugal casting, after cyclic oxidation at (**a**) 800 °C and (**b**) 1000 °C for 400 h.

**Figure 12:**
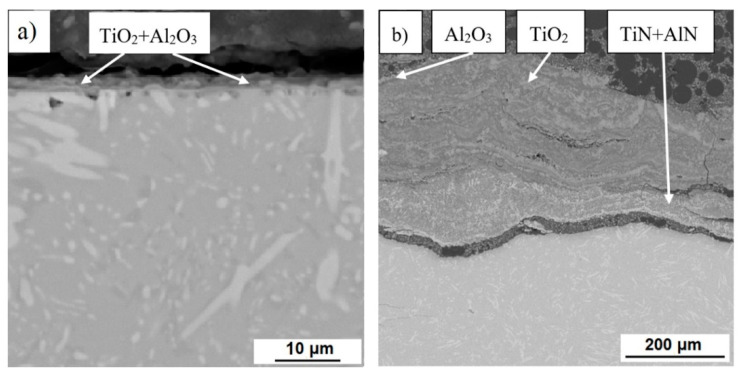
Microstructure (SEM-BSE) of oxide layers of TiAl35Si5 alloy, prepared by centrifugal casting followed by hot isostatic pressing, after cyclic oxidation at (**a**) 800 °C and (**b**) 1000 °C for 400 h.

The microstructures of the oxide layers of all studied alloys after cyclic oxidation tests at 800 °C and 1000 °C are shown in Figure 10, Figure 11 and Figure 12. The surface oxide layer of cast alloys basically consisted of two layers. The upper layer consisted largely of titanium dioxide TiO_2_ and the sublayer of alumina Al_2_O_3_ and a mixture thereof in various proportions. In the very thin layer after oxidation at 800 °C, which formed in TiAl35Si5 (CC+HIP), only a mixture of these oxides was detected by EDS analysis (see Figure 12). At lower oxidation temperatures, a significantly thinner layer of formed oxides can be observed on cast alloys compared to higher temperatures. In cast TiAl35Si5 alloys, this layer well protected the material from further oxidation. After oxidation at 1000 °C, there was a large increase in the oxide layer of all cast alloys and its delamination, specifically in TiAl35Si5 alloys (see Figure 11), due to poor adhesion. In the case of the TiAl15Si15 alloy, phases of less brittle titanium silicides TiSi2 and TiSi were also detected (Figure 10), which most likely prevented delamination of the porous oxide layer even at a higher oxidation temperature, but the material was completely oxidized.

## 4. Discussion

The Ti-Al-Si alloys developed at UCT Prague have been manufactured by the methods of powder metallurgy, i.e., by the means of a reactive sintering and a combination of mechanical alloying and spark plasma sintering. While the first method enables very simple synthesis, but highly porous products, the second one allows to obtain low-porosity compacts with ultrafine-grained microstructure. This nanomaterial reaches very high compressive strength (up to 2300 MPa) [31], but also very low room-temperature toughness and negligible ductility. This could be attributed to one of the following phenomena: blocking of dislocation movement by grain boundaries in fine structure, high residual internal stresses after mechanical alloying, which do not relax during reactive sintering or high brittleness of titanium silicide particles. In our recent work on cast Ti-Al-Si materials, where the fracture toughness has been studied, we found that the cracks initiate in the brittle silicide phase and stop in more ductile aluminide phase.Therefore, the improvement of fracture toughness can be probably achieved by coarser microstructure of the alloy, where larger areas of aluminide phase are present.

From the viewpoint of the wear resistance, the lower-silicon alloy was identified as the more advantageous one, even though its hardness is lower. The reason is probably in the higher amount of more ductile aluminide phase, because the hard and brittle silicide phase tends to crack and remove from the wear track during the wear test. The influence of HIP treatment on the wear behavior, ultimate compressive strength and hardness was negligible, even though this process visibly changed the morphology of the silicide phase. In the steel, such spheroidization of carbides decreases the hardness, as it practically applied in soft annealing process [32]. However, it can be highly expected that the change of the morphology of silicide particles to round ones will strongly affect the toughness and fatigue life due to minimization of the stress concentration at the edges of the silicide particles.

Temperatures of 800 °C and 1000 °C were chosen for cyclic oxidation tests. The first of the temperatures was selected on the basis of knowledge of the usability temperature limit of intermetallic compounds based on the Ti-Al system [25]. Therefore, due to the assumption of a positive effect of silicon in the alloys, the second temperature was raised to 1000 °C. Cyclic oxidation tests were performed for 400 h with a cycle of 50 h and were used to detect the susceptibility of the formed oxide layers to delamination due to thermally induced stress. They have also been applied as a result of simulating the environment to which these alloys could be exposed in the future, for example in aircraft jet engines. The results indicate that the alloy TiAl15Si15 processed by casting is not sufficiently oxidation resistant under given conditions, because the oxide layer at 800 °C is porous, and 1000 °C leads to complete oxidation of the material. It can be caused by carbon contamination of the alloy, where the carbon could be oxidized in near-surface areas during the test, leaving the pores in the material, as it is visible in Figure 11a. Generally, the results of the oxidation resistance are worse than in fine-grained materials prepared by mechanical alloying [28]. The probable reason is that the silicon, which improves the adherence of the oxide scales [33], is more homogenously distributed in the alloy when the mechanical alloying is used. It is due to finer and homogeneously dispersed particles of titanium silicide and also thanks to the supersaturation of the aluminide matrix by silicon. Hence, the coarse and equilibrium structures of cast alloys are not so advantageous for high-temperature service from the viewpoint of the oxidation resistance. The result also proved the recently proposed mechanism of the influence of silicon on the oxidation resistance [34], where silicon is concentrated below the oxide layer in the form of silicides (here Ti_5_Si_3_ and also TiSi_2_, which was not present in the alloy before the oxidation), while aluminum and titanium form oxides in the oxide layer. The silicide layer acts as the secondary protection against the oxidation.

Compared to conventionally used materials (steels, nickel alloys), Ti-Al-Si alloys have a significantly lower density, but the problem is cost and difficult production. The abrasion wear resistance of Ti-Al-Si alloys is comparable to tool steels, but Ti-Al-Si alloys have the potential advantage of higher resistance to elevated temperatures during high-speed machining over steels if potentially used in tool producing. In addition, additional heat treatment is required for steels to achieve these properties, while not for Ti-Al-Si materials.

## 5. Conclusions

The Ti-Al-Si alloys processed by centrifugal casting exhibit the microstructure composed of titanium aluminide (TiAl) matrix and hard and brittle sharp-edged Ti_5_Si_3_ particles. The morphology of silicide particles can be transformed to rounded ones by the application of HIP. TiAl35Si5 alloy has been shown to be sufficiently resistant to high temperature oxidation at 800 °C. However, at an elevated temperature of 1000 °C, the protective effect of the layer was lost, and its massive delamination occurred. The TiAl15Si15 alloy is characterized by poor oxidation and wear resistance, probably due to coarse silicide particles and carbon contamination from the processing. The results proved the influence of silicon on the oxidation mechanism in the form of a silicide layer below the oxide scales, which acts as the secondary barrier against the diffusion of oxygen and oxidized metals.

## Figures and Tables

**Table 1:** Mechanical properties of tested alloys.

Mechanical Properties	TiAl15Si15 (CC)	TiAl35Si5 (CC)	TiAl35Si5 (CC + HIP)
Hardness HV 5	459 ± 15	375 ± 16	374 ± 7
Ultimate compressive strength [MPa]	1205 ± 80	1867 ± 75	1801 ± 47

**Table 2:** Tribological properties of tested alloys.

Tribological Properties	TiAl15Si15 (CC)	TiAl35Si5 (CC)	TiAl35Si5 (CC + HIP)
friction coefficient [–]	0.555 ± 0.006	0.453 ± 0.004	0.462 ± 0.003
wear rate [mm^3^m^−1^N^−1^]	(4.17 ± 0.09) × 10 ^−4^	(1.25 ± 0.02) × 10 ^−4^	(1.21 ± 0.04) × 10 ^−4^

## Data Availability

The data are stored by the authors of the paper, not available publically.
